# Worries, Preparedness, and Perceived Impact of Covid-19 Pandemic on Nurses' Mental Health

**DOI:** 10.3389/fpubh.2021.566700

**Published:** 2021-05-26

**Authors:** Maura Galletta, Ilenia Piras, Gabriele Finco, Federico Meloni, Ernesto D'Aloja, Paolo Contu, Marcello Campagna, Igor Portoghese

**Affiliations:** ^1^Department of Medical Sciences and Public Health, University of Cagliari, Cagliari, Italy; ^2^PhD School in Biomedical Sciences (Public Health), University of Sassari, Sassari, Italy; ^3^Emergency Department SS. Trinità Hospital, Azienda Tutela Salute Sardegna, Cagliari, Italy; ^4^Pain Therapy Service, University of Cagliari, Cagliari, Italy

**Keywords:** COVID-19 pandemic, nurses, stress, health outcomes, risk factors, perceived impact

## Abstract

**Background:** In times of global public health emergency, such as the COVID-19 pandemic, nurses stand at the front line, working in close contact with infected individuals. Being actively engaged in fighting against COVID-19 exposes nurses to a high risk of being infected but can also have a serious impact on their mental health, as they are faced with excessive workload and emotional burden in many front-line operating contexts.

**Purpose:** The aim of the study is to analyze how risk factors such as perceived impact, preparedness to the pandemic, and worries were associated with mental health outcomes (crying, rumination and stress) in nurses.

**Methods:** A cross-sectional study design was performed via an online questionnaire survey. Participants included 894 registered nurses from Italy. Participation was voluntary and anonymous. Multiple binary logistic regression was carried out to analyze the relationship between risk factors and health outcomes.

**Results:** Increased job stress was related to higher levels of rumination about the pandemic (OR = 4.04, *p* < 0.001), job demand (OR = 2.00, *p* < 0.001), impact on job role (OR = 2.56, *p* < 0.001), watching coworkers crying at work (OR = 1.50, *p* < 0.05), non-work-related concerns (OR = 2.28, *p* < 0.001), and fear of getting infected (OR = 2.05, *p* < 0.001). Job stress (OR = 2.52, *p* < 0.01), rumination (OR = 2.28, *p* < 0.001), and watching colleagues crying (OR = 7.92, *p* < 0.001) were associated with crying at work. Rumination was associated with caring for patients who died of COVID-19 (OR = 1.54, *p* < 0.05), job demand (OR = 1.70, *p* < 0.01), watching colleagues crying (OR = 1.81, *p* < 0.001), non-work-related worries (OR = 1.57, *p* < 0.05), and fear of getting infected (OR = 2.02, *p* < 0.001).

**Conclusions:** The psychological impact that this pandemic may cause in the medium/long term could be greater than the economical one. This is the main challenge that health organizations will have to face in the future. This study highlights that the perceived impact and worries about the pandemic affect nurses' mental health and can impact on their overall effectiveness during the pandemic. Measures to enhance nurses' protection and to lessen the risk of depressive symptoms and post-traumatic stress should be planned promptly.

## Introduction

The Coronavirus Disease (COVID-19) pandemic represents a serious concern for public and occupational health (1). This pandemic is having an unprecedented impact on the nursing profession. According to the World Health Organization, nurses represent the largest group of Health Care Workers (HCWs) involved on the front line of health care systems. In this sense, nurses deliver care to patients in close physical proximity and thus they are directly exposed to the virus and are at high risk of developing the disease (2–4). To protect HCWs, physical distancing in taking care of COVID-19 patients can limit the spread of the infection, although it reduces nurses' ability to meet the patients' needs. However, during the first months of the pandemic, the European Center for Disease Control and Prevention (ECDC) (5) announced that up to 10% of the reported cases in China and up to 9% of all cases in Italy were among HCWs. According to the CDC (6) in the US the percentage of positive cases among HCWs ranged from 3 to 11%. However, due to the preventive measures, the infection risk among HCWs gradually decreased (7). What rapidly became important was to preserve the mental health of HCWs (8), challenged by the tremendous psychosocial crisis they were experiencing (9–11).

In pandemic scenarios, all HCWs are at risk of long working hours, higher job demands, psychological distress, fatigue, stigmatization, and physical and psychological violence (2). Studies showed the impact of this critical situation on HCWs' mental health in terms of worries, fatigue, insomnia, anxiety, depression, and stress (8, 11–13). Moreover, the increased percentage of patient deaths results in an augmented exposure to emotional and psychological suffering: a recent systematic review on HCWs' mental health during the COVID-19 pandemic found an anxiety incidence of 24.6%, a depression incidence of 22.8%, and an insomnia incidence of 34.3% (14). Regarding the psychological impact of the outbreak, the literature points out a prevalence of post-traumatic stress disorder (PTSD) among HCWs between 11 and 73.4% (15). The continuous exposure to stressful events may result in post-traumatic stress symptoms, which, in turn, may mine professionals' ability to cope with the situation. As to the nursing profession, previous studies investigating the impact of outbreaks/pandemic showed that the more nurses perceived risks for their health, the more they left their job (16–18). Furthermore, those who did not leave were exposed by the pandemic scenario to higher levels of distress, increased workload, emotional burden, workplace conflicts, increased depression risk, and suicide (19, 20). In a recent literature review investigating the impact of respiratory pandemic on nurses, Fernandez et al. (21) reported that nurses experienced fear, worries for personal and family safety, a sense of powerlessness, increased job demands, anxiety, and stress. Furthermore, perceived organizational preparedness and safety played a crucial role.

In this sense, preserving nurses' mental health during the COVID-19 pandemic is a very important global challenge as it may increase health systems' ability to deliver timely care. Worries and emotional impact of the COVID-19 pandemic among nurses are still barely analyzed. Most of the available studies on the topic usually include physicians or other health care professionals (22). In relation to the peculiarities of its professional mandate and the current organization of the Italian health care service, the nursing profession is facing this critical situation in a transversal way, in different care contexts. Moreover, nurses are faced with an excessive workload and emotional burden in many front-line operating realities, even compared to the actual available resources (23). In Italy, in the first months of the pandemic, 25,629 health workers were infected with Covid-19 (24), including 12,000 nurses (25). In addition, of the 80 health workers who died (16), 39 were nurses, four of whom committed suicide (25). To our knowledge, there is no Italian study analyzing nurses' mental health perception during this pandemic, because most of the COVID-19-related research includes Asian samples (26). Therefore, this study would contribute to expand the knowledge on the topic and provide additional value to the existing studies.

## Study Aim

The aim of the study was to analyze how the perceived impact, preparedness to the pandemic, and worries are associated with mental health indicators (crying, rumination, and stress) in nurses.

## Materials and Methods

### Study Design, Participants, and Data Collection

A cross-sectional study design was performed via an online questionnaire survey. Participants included registered nurses from Italy. The only inclusion criterion was to be working during the COVID-19 pandemic. To collect data, the LimeSurvey application was implemented and the link to the questionnaire was shared through social networking platforms. Participants were briefed about the study purpose through written information reported on the questionnaire's homepage. Informed consent was obtained from all nurses before filling out the online questionnaire. Privacy was assured because no IP address was registered and no sensitive data were requested. The data were collected from April 15th to April 24th 2020.

### Ethical Statement

The study complies with the Declaration of Helsinki and with the General Data Protection Regulation (EU) 2016/679 (GDPR). Participation was voluntary and anonymous, according to Italian Data protection law (e.g., Decree n. 196/2003). Participants could interrupt their participation in the survey at any time without any adverse consequence. We consulted the Institutional Review Boards of the University of Cagliari, which informally said that Ethical review and approval was not required for the study on human participants, in accordance with the local legislation and institutional requirements.

### Measures

The online self-report questionnaire consisted of two sections. The first one regarded demographic information including gender, age, working geographical area, civil status, children, current clinical-healthcare area, job description, and professional tenure. The second one was developed by combining items from different questionnaires. Specifically, we used measures from previous international studies on other epidemics (SARS and Avian Influenza) (27, 28) to assess worries, preparedness and impact of the COVID-19 pandemic among nurses. The aim was to use a short survey to avoid cognitive overloading for workers. In this sense, items unsuitable for the target work population were not selected. Also, we used Rumination on Sadness Scale (29, 30) to measure nurses' rumination about the pandemic. Finally, we measured the frequency of crying at work and watching one's own colleagues crying at work. For the scales that did not have an Italian version, cultural adaptability of the items was assured via translation and back-translation procedures (31). Two bilingual experts independently translated the questionnaire from English into Italian. The two translations were then compared to identify and discuss the main inconsistencies. After this revision, a final Italian version of the questionnaire was created. Then, the translated questionnaire was back-translated into English by another bilingual linguistic expert to evaluate equivalence. Finally, the back-translated version of the questionnaire was compared with the original version. Meanings and concepts were considered as equivalent. A pre-test was carried out on 10 nurses to assess the appropriateness of the translation, comprehensibility and clarity of the items, and time of completing questionnaire.

Regarding the specific measures considered into the whole questionnaire, we investigated the following variables: (1) organizational preparedness (1 item) and Regional Health System (RHS) preparedness (3 items). A sample item was “My hospital RHS has a preparedness plan for the COVID-19 pandemic”; (2) personal preparedness (1 item: “I am personally prepared for the COVID-19 pandemic”); (3) fear of getting sick with COVID-19 (2 items: “I am afraid of falling ill with COVID-19”); (4) non-work-related concerns (3 items: “People close to me are at high risk of getting COVID-19 because of my job”); (5) increased job demands (3 items: “I had an increase in workload in my job”) and job role (1 item: “I would had to do work not normally done by me”); (6) impact on personal life (3 items: “People avoid me because of my job”); (7) perceived job stress (1 item: “I feel more stressed at work”). Rumination about the pandemic was measured by adapting 2 items from the Italian version of Rumination on Sadness Scale changing “sadness” with “pandemic” (e.g., “I have difficulty getting myself to stop thinking about this pandemic”) (29, 30). Finally, 2 items were *ad hoc* developed to measure the frequency of crying at work and watching one's own colleagues crying at work (e.g., “I have been crying at work because I felt like I could not take it anymore”). Cronbach's Alpha was calculated for the scales with at least three items. For the measures with two items, inter-item correlation was performed. All the items were based on a five-point Likert scale ranging from 1 (strongly disagree) to 5 (strongly agree).

### Statistical Analysis

To performing data analysis, the SPSS (IBM, Chicago, IL, USA) version 23.0 was used. Descriptive analyses such as frequencies, percentages, mean and standard deviation, and median and interquartile range (IQR) were carried out to analyze the descriptive characteristics of the sample for the study variables. Multi-item scores were computed by calculating the mean of the items in each scale. All the study variables were divided in low/high rate for the variable. The central point (=3) of the rating scale was considered as the cut-off criteria: the values ≤ 3 were rated as 0 (low) and those ≥3 were rated as 1 (high). Mann–Whitney *U* and Pearson chi-square (χ^2^) tests were performed to compare sub-groups of the sample by discriminating for work context (frontline/non-frontline) and for presence/absence of patients who died of COVID-19. Frequencies and percentages for the variables regarding the work history (working geographical area, working area, and professional tenure) and demographic characteristics (gender, age, civil status, and children) of the participants, were compared to detect possible differences between groups. Also, we explored differences between groups of age (≤ 45 and >45 years old), family status (single, conjugate, divorced, widower, and other), work geographical area (North-Center and South-Islands), and work context (frontline/non frontline) with regard to social ostracism (low/high), non-working concerns (low/high), concern for friends (non-worried/worried), concern for colleagues (non-worried/worried), concern for patients (non-worried/worried). Cut-off for age was defined based on sample distribution in percentiles, namely considering all the individuals who were below and above the 50° percentile. Crying at work, rumination, and perceived stress were identified as potential risk outcomes for health among nurses. Perceived impact, preparedness for the pandemic, and worries were considered as main risk factors. To analyze the risk factors on health outcomes, multiple binary logistic regression was carried out by reporting odds ratios (ORs) and 95% Confidence Intervals (CI). The model was adjusted for gender, age, frontline/non-frontline nurse, and caring/non-caring for patients who died of COVID-19. These variables were considered as potential confounders. The significance level was set at *p* = 0.05.

About the instrument reliability, Cronbach's alpha coefficients for RHS preparedness was 0.80, for non-work-related concerns was 0.66, for increased job demands was 0.74, and for perceived impact on personal life was 0.74. Inter-item correlations were all significant at *p* < 0.001. Specifically, inter-item correlation for fear of getting sick with COVID-19 was 0.37, for rumination about the pandemic was 0.76, and for crying at work was 0.52.

## Results

A total of 894 nurses completed the questionnaire. However, 34 nurses who were not actively at work at the time of the study had to be excluded from it. Thus, the final sample for this study consisted of 860 nurses, most of whom were women (77.7%, *n* = 668). Regarding age, the larger proportion ranged from 50 to 59 years (32%, *n* = 275). The majority of respondents worked in southern Italy or on one of the Islands (60.3%, *n* = 519), had an average professional tenure of 18.5 years (SD = 11.6), worked in hospital context (79.5%, *n* = 684), had children (51.6%, *n* = 444), and was married (43.7%, *n* = 376). Furthermore, 44.3% (*n* = 381) were frontline nurses working in a COVID-19 emergency unit. Finally, 32.7% of nurses (*n* = 281) attended training courses and 17.2% (*n* = 148) attended audits on infection control in the 6 months before the pandemic; 26.7% (*n* = 230) purchased personal protective equipment; 34.7% (*n* = 298) cared for patients who died of COVID-19.

### Nurses' Descriptive and Job Characteristics

Tables 1, 2 show both the demographic and the job characteristics of the sample. We split the sample into frontline/non-frontline nurses and caring/non-caring for patients who died of COVID-2019. Specifically, Table 1 shows significant differences between frontline and non-frontline nurses for all the variables in the analysis (Gender: χ^2^ = 6.06, *p* = 0.014; Median age: Mann–Whitney *U* = 74.26, *p* < 0.001; Age range: χ^2^ = 29.03, *p* < 0.001; Working geographical area: χ^2^ = 66.09, *p* < 0.001; Civil status: χ^2^ = 13.45, *p* = 0.009; Children: χ^2^ = 5.25, *p* < 0.022; Working area: χ^2^ = 70.06, *p* < 0.001; Professional tenure: Mann–Whitney *U* = 78.68, *p* = 0.001). Table 2 shows significant differences between nurses who cared and those who did not care for patients who died of COVID-2019 for age (Median age: Mann–Whitney *U* = 68.04, *p* < 0.001; Age range: χ^2^ = 22.42, *p* < 0.001), working geographical area (χ^2^ = 173.20, *p* < 0.001), working area (χ^2^ = 46.40, *p* < 0.001), and professional tenure (Mann–Whitney *U* = 70.65, *p* < 0.001).

**Table 1:** Differences between frontline and non-frontline nurses on both demographic and job characteristics.

**Variable**	**Non-frontline nurse**	**Frontline nurse**	***p*-value**
Gender			
Male, *n* (%)	92 (19.2)	100 (26.2)	0.014^a^
Woman, *n* (%)	387 (80.8)	281 (73.8)	
Median age (IQR)	48.0 (33.0–60.0)	43.0 (32.0–57.0)	<0.001^b^
Age (range)			
<30 years, *n* (%)	73 (15.2)	69 (18.1)	<0.001^a^
30–39 years, *n* (%)	89 (18.6)	98 (25.7)	
40–49 years, *n* (%)	107 (22.3)	113 (29.7)	
50–59 years, *n* (%)	183 (38.2)	92 (24.1)	
≥60 years, *n* (%)	27 (5.6)	9 (2.4)	
Working geographical area			
Northern or Central Italy, *n* (%)	132 (27.6)	209 (54.9)	<0.001^a^
Southern Italy or Islands, *n* (%)	347 (72.4)	172 (45.1)	
Family status			
Single, *n* (%)	176 (36.7)	171 (44.9)	0.009^a^
Married, *n* (%)	231 (48.2)	145 (38.1)	
Divorcee, *n* (%)	29 (6.1)	34 (8.9)	
Widower, *n* (%)	6 (1.3)	1 (0.3)	
Other, *n* (%)	37 (7.7)	30 (7.9)	
Children			
Yes *n* (%)	264 (55.1)	180 (47.2)	0.022^a^
No *n* (%)	215 (44.9)	201 (52.8)	
Workplace area			
Hospital, *n* (%)	334 (69.7)	350 (91.9)	<0.001^a^
Territorial service, *n* (%)	81 (16.9)	27 (7.1)	
Other, *n* (%)	64 (13.4)	4 (1.0)	
Professional tenure (median and IQR)	24.0 (7.0–37.0)	16.0 (7.0–35.0)	0.001^b^

*IQR, Interquartile Range. ^a^Pearson chi-square test. ^b^Mann–Whitney U-test*.

**Table 2:** Differences between nurses who cared for and nurses who did not care for patients who died of COVID-19 on both demographic and job characteristics.

**Variable**	**Non-caring for patients who died of COVID-19**	**Caring for patients who died of COVID-19**	***p*-value**
Gender			
Male, *n* (%)	114 (20.3)	78 (26.2)	0.048^a^
Woman, *n* (%)	448 (79.7)	220 (73.8)	
Median age (IQR)	47.5 (34.0–60.0)	42.5 (32.0–56.0)	<0.001^b^
Age (range)			
<30 years, *n* (%)	84 (14.9)	58 (19.5)	<0.001^a^
30–39 years, *n* (%)	114 (20.3)	73 (24.5)	
40–49 years, *n* (%)	130 (23.1)	90 (30.2)	
50–59 years, *n* (%)	204 (36.3)	71 (23.8)	
≥60 years, *n* (%)	30 (5.3)	6 (2.0)	
Working geographical area			
Northern or Central Italy, *n* (%)	133 (23.7)	208 (69.8)	<0.001^a^
Southern Italy orIslands, *n* (%)	429 (76.3)	90 (30.2)	
Family status			
Single, *n* (%)	219 (39.0)	128 (43.0)	0.158^a^
Married, *n* (%)	260 (46.3)	116 (38.9)	
Divorcee, *n* (%)	37 (6.6)	26 (8.7)	
Widower, *n* (%)	6 (1.1)	1 (0.3)	
Other, *n* (%)	40 (7.1)	27 (9.1)	
Children			
Yes, *n* (%)	302 (53.7)	142 (47.7)	0.089^a^
No, *n* (%)	260 (46.3)	156 (52.3)	
Working area			
Hospital, *n* (%)	409 (72.8)	275 (92.3)	<0.001^a^
Territorial service, *n* (%)	91 (16.2)	17 (5.7)	
Other, *n* (%)	62 (11.0)	6 (2.0)	
Professional tenure (median and IQR)	23.5 (8.0–37.0)	15.0 (6.0–35.0)	<0.001^b^

*IQR, Interquartile Range. ^a^Pearson chi-square test. ^b^Mann–Whitney U-test*.

### Worries, Preparedness, and Perceived Impact of the COVID-19 Pandemic

Regarding worries about the pandemic, 73.3% (*n* = 630) of nurses was afraid of getting sick with COVID-19, and 73.7% (*n* = 634) was concerned about putting their family at risk of getting infected. Regarding nurses' preparedness, 79.9% (*n* = 687) of respondents did not feel prepared for the pandemic. In fact, a small percentage (18.3%, *n* = 157) of the sample declared to have received adequate training regarding COVID-19, and 34.3% (*n* = 295) referred to have received adequate training and support on personal protective equipment (PPE). Ninety-three percent (*n* = 799) of nurses referred that their organization was not prepared for COVID-19 pandemic, and 91.0% (*n* = 783) felt that the RHS was not prepared for the pandemic. Seven hundred seventy-nine (90.6%) participants declared that RHS did not inform them about the pandemic management plan.

With regard to the perceived impact of the pandemic on job duties, 46.2% (*n* = 397) of nurses referred increased job demands, and 39.2% (*n* = 337) declared to have carried out tasks outside of their daily duties. Regarding the perceived impact on personal life, 25.0% (*n* = 215) of the participants referred to have been avoided by other people because of their job; 12.2% (*n* = 105) declared that their families were avoided as well because of their job; 8.8% (*n* = 76) referred to avoid telling people about the nature of their job. With regard to non-work-related worries, 63.5% (*n* = 546) of nurses felt that their job would cause their loved ones to run a high risk of COVID-19 infection; 72% (*n* = 619) of nurses reported that their own health was a cause of worry for their loved ones, and 56.0% (*n* = 482) reported that their loved ones were worried to be infected by them. Moreover, results highlight that nurses were fairly concerned for their close friends (29.3%, *n* = 252), for their colleagues (38.1%, *n* = 328), and for their patients (28.5%, *n* = 245). They were very concerned for their partner (25.6%, *n* = 220) and extremely concerned for their children (21.4%, *n* = 184), parents (35.3%, *n* = 304), and old relatives (31.2%, *n* = 268). Regarding health results, 66.0% (*n* = 568) of the participants felt more stressed because of the pandemic, 44.0% (*n* = 378) declared to have a high level of rumination about the pandemic, 19.9% (*n* = 171) referred to have cried at work, and 34.5% (*n* = 296) reported to have watched colleagues crying at work. Furthermore, we compared the study variables discriminating by age, family status, region, and working context. Regarding differences between age ranges, the results showed that young nurses perceived higher non-work-related concerns about infecting family members (79.5%) than elderly nurses (67.8%) (χ^2^ = 15.07, *p* < 0.001). Moreover, younger nurses were more worried for both their colleagues' health (79.1%) and their patients' health (84.0%) (χ^2^ = 9.70, *p* < 0.01) than older nurses (69.7 and 77.3%, respectively) (χ^2^ = 5.76, *p* < 0.05). Regarding differences in terms of family status, single and divorced nurses are more worried for their friends (73.4 and 61.8%, respectively) (χ^2^ = 22.86, *p* < 0.001) and colleagues (80.5 and 75.8%, respectively) (χ^2^ = 14.99, *p* < 0.01) than nurses with a different family status (conjugate, widower, and other). Regarding work context, frontline nurses registered higher levels of perceived ostracism (16%) than non-frontline nurses (10.6%) (χ^2^ = 5.39, *p* < 0.05), and higher non-work-related worries (78.0 and 70.4%, respectively) (χ^2^ = 6.32, *p* < 0.05). No significant differences were found between nurses working in different geographical areas (North-Center and South-Islands) for the study variables.

### Relationships Between Worries, Preparedness, and Perceived Impact of the Pandemic on Health Results

Table 3 presents the results of multivariate analyses. Three binary logistic regression models were performed:the demographic variables included gender, age, frontline/non-frontline nurse, and caring/non-caring for patients who died of COVID-19. The first model included perceived job stress as a dependent variable. The results showed that increased job stress was significantly related to a higher level of rumination (OR = 4.04, 95% CI = 2.77–5.89, and *p* < 0.001), increased job demand (OR = 2.00, 95% CI = 1.38–2.90, and *p* < 0.001), impact on one's job role (OR = 2.56, 95% CI = 1.74–3.77, and *p* < 0.001), watching coworkers crying at work (OR = 1.50, 95% CI = 1.01–2.22, and *p* < 0.05), non-work-related concerns (OR = 2.28, 95% CI = 1.54–3.38, *p* < 0.001), and worry about getting infected (OR = 2.05, 95% CI = 1.39–3.01, and *p* < 0.001). Among control variables, gender was significantly associated with job stress (OR = 1.71, 95% CI = 1.14–2.56, and *p* < 0.05). Specifically, women are more vulnerable to higher levels of stress. Age, caring for patients who died of COVID-19, and being a frontline nurse did not affect perceived job stress. The second regression model included crying at work as a health outcome for nurses. The results showed that increased job stress (OR = 2.52, 95% CI = 1.39–4.56, and *p* < 0.01), rumination on pandemic (OR = 2.28, 95% CI = 1.48–3.51, and *p* < 0.001), and watching colleagues crying at work (OR = 7.92, 95% CI = 5.16–12.16, and *p* < 0.001) were the predictors significantly associated with crying at work. Among the demographic variables, gender was significantly associated with crying at work (OR = 4.61, 95% CI = 2.40–8.86, and *p* < 0.001): females were used as a referral for the regression analysis as well, and the results show that women cry more than men. The third model included rumination on the pandemic as a dependent variable. The results showed that higher levels of rumination were associated with caring for patients who died of COVID-19 (OR = 1.54, 95% CI = 1.07–2.21, and *p* < 0.05), increased job demand (OR = 1.70, 95% CI = 1.25–2.33, and *p* < 0.01), watching colleagues crying at work (OR = 1.81, 95% CI = 1.32–2.48, and *p* < 0.001), non-work-related worries (OR = 1.57, 95% CI = 1.08–2.30, and *p* < 0.05), and worries about getting infected (OR = 2.02, 95% CI = 1.39–2.93, and *p* < 0.001). Gender and age were both significant (OR = 1.45, 95% CI = 1.01–2.07, and *p* < 0.05; OR = 1.32, 95% CI = 1.16–1.50, and *p* < 0.001, respectively). Regarding gender, women are more inclined to rumination than men. Regarding age, older nurses are more ruminative than younger ones.

**Table 3:** Binary logistic regression results: relationship between worries, preparedness, and impact of the pandemic on nurses' health outcomes.

	**Perceived job stress**			**Crying at work**			**Rumination about pandemic**		
**Variables in the equation**	**OR**	**95% CI for OR**		**OR**	**95% CI for OR**		**OR**	**95% CI for OR**	
		**Lower**	**Upper**		**Lower**	**Upper**		**Lower**	**Upper**
Frontline/non-frontline	0.858	0.573	1.284	1.138	0.713	1.816	1.063	0.751	1.504
Gender	**1.708**	**1.145**	**2.547**	**4.608**	**2.396**	**8.863**	**1.447**	**1.009**	**2.075**
Age	1.082	0.931	1.256	1.131	0.945	1.354	**1.320**	**1.158**	**1.505**
Dead patients (yes/no)	0.859	0.555	1.331	1.032	0.637	1.673	**1.540**	**1.071**	**2.214**
Perceived job stress	–	–	–	**2.516**	**1.387**	**4.564**	–	–	–
Rumination	**4.041**	**2.772**	**5.890**	**2.279**	**1.479**	**3.511**	–	–	–
Increased job demand	**2.002**	**1.382**	**2.901**	1.526	0.987	2.360	**1.705**	**1.246**	**2.334**
Impact on job role	**2.559**	**1.737**	**3.769**	1.152	0.751	1.767	1.048	0.762	1.442
Watching colleagues crying at work	**1.501**	**1.014**	**2.222**	**7.924**	**5.165**	**12.157**	**1.808**	**1.320**	**2.478**
Impact on personal life (social ostracism)	1.322	0.744	2.349	1.141	0.667	1.954	1.543	0.995	2.393
Non-work-related worries	**2.281**	**1.540**	**3.379**	1.335	0.743	2.401	**1.575**	**1.081**	**2.296**
Worry about getting infected	**2.049**	**1.392**	**3.015**	1.513	0.849	2.698	**2.022**	**1.394**	**2.932**
Personal preparedness	0.918	0.584	1.443	0.941	0.536	1.652	0.792	0.531	1.183
Adequate support and Info about PPE	0.844	0.576	1.237	1.060	0.668	1.682	0.981	0.703	1.369
RHS preparedness	0.751	0.394	1.431	1.936	0.879	4.264	1.002	0.556	1.803
Organization preparedness	0.861	0.404	1.836	0.488	0.196	1.216	1.900	0.994	3.630

*OR, Odd ratio; CI, confidence interval, Values in bold denote significance*.

## Discussion

Investigating HCWs' perceived impact and worries on the COVID-19 pandemic is crucial to safeguard professionals' mental health.

The results showed that younger nurses reported higher worries about infecting their family members than older nurses, as well as higher worries about their colleagues and patients' health. Therefore, as a result of this emotional state, younger nurses might be at greater risk for developing stress (13), thus suggesting healthcare organization should pay attention to safeguarding young nurses during this pandemic. Moreover, frontline nurses perceived higher levels of perceived ostracism than non-frontline nurses due to their close contact with patients affected by the virus and high worries about infecting their families and loved ones. Stigmatization and ostracism are aspects that also emerged in recent studies (11, 32) and previous outbreaks (27). These factors emerged to be negatively related to nurses' mental health and stress (33).

With regard to the perceived impact of the COVID-19 pandemic on job duties, 46% of nurses reported increased job demands during the emergency. This aligns with literature that emphasized augmented workload during pandemics (34). Regarding the perceived impact on personal life, about 46% of nurses reported that people avoided them ortheir families because of their job. This is quite consistent with a previous study developed during the SARS virus in which health care providers experienced discrimination during the epidemic (28). As to non-work-related worries, 64% of nurses were concerned about putting their loves ones' life at risk. Analogously with previous studies (35), they were mostly worried about their partner, children, parents, and old relatives. Thus, ensuring quarantine to professionals who work with COVID-19 patients would be important to strengthen safety-feeling among nurses. Previous studies showed that the main emotional response to the epidemic/pandemic is increased job stress (36, 37). Our findings support those results by revealing that 66% of nurses perceived high level of stress. In addition, 44.0% of the Italian nurse sample declared to have had higher levels of ruminative thinking about the pandemic. Although there is no study on rumination in HCWs during outbreaks/pandemics, literature shows that rumination is associated with greater burnout, depression, and risk of psychiatric morbidity (38). Rumination is a frequent automatic and passive cognitive activity: people with ruminative thinking tend to remain fixated on the problems without taking action (39). As a result, this dysfunctional response style may compromise emotional processes and negatively influence nurses' mental health (40), as well as hindering an individual's goal achievement (41). About 20% of nurses stated to have cried at work, and 34% declared to have watched their colleagues crying. Crying is a signal that typically communicates emotional distress and is an important symptom that indicates the difficulty to manage work-related emotional pressures (42). For this reason, crying should be considered as a sign of nurses' mental health.

The analysis of the relationship between variables on the three health outcomes (job stress, crying at work, and rumination) showed that worries about getting infected, increased job demand, impact on job role, non-work-related worries, watching colleagues crying, and ruminative thinking were significantly associated with perceived job stress. Moreover, rumination, job stress, and watching colleagues crying were the risk factors associated with crying at work among nurses. Finally, job demands, non-work-related worries, worries about getting infected, and watching colleagues crying were the main factors associated with rumination about the pandemic. Overall, these results are in line with previous research showing that HCWs experienced increased stress through infectious epidemics (34, 43). According to these studies, we found that worries about falling ill with COVID-19 and putting nurses' loved ones at risk were the main sources of stress. Although, in line with previous research (34, 43), this study showed that the impact of the pandemic on personal life (social ostracism), personal preparedness, RHS and workplace preparedness, and adequate support and information about PPE are important safety aspects for nurses which are not significantly associated with health outcomes. They could likely have an indirect effect on health outcomes, but worries are the main factor that may affect nurses' perceived effectiveness in the pandemic. As a result, pandemics increases nurses' workload due to the increased number of patients to care for, prolonged working hours, and working on tasks that they normally do not perform (27, 44), thus increasing perceived job stress. In addition, the important demands that nurses have to face during the pandemic usually add further emotional requests. Continuous exposure to patients' death and suffering can lead to vicarious trauma and secondary traumatic stress (12, 45). Furthermore, our study shows that crying at work is associated with higher levels of job stress and rumination about the pandemic. This can be due to excessive demands and emotional pressures (42, 46) perceived by nurses during the pandemic. Very interesting is also the role of emotional contagion in watching colleagues crying at work, which would result in a worsening of the symptoms probably due to the shared psychological environment. Finally, increased job stress is associated with rumination about the COVID-19 pandemic, whose main factors are job demands, working and non-work-related concerns, and watching colleagues crying at work. Previous research suggests that person-directed interventions such as cognitive behavioral therapy and relaxation exercises would effectively decrease ruminative thinking (38, 47) and protect nurses' wellbeing. Among covariates, our findings show no difference between frontline or non-frontline nurses in terms of health results. Therefore, although previous studies on epidemics (SARS) paid attention especially to frontline HCWs as professionals at risk of developing traumatic stress (36, 48), in this study we found that being a frontline nurse is not a significant health risk factor. This suggests that the mental health of all professionals from any clinical-care context is at risk during the pandemic. In fact, a recent study on COVID-19 pandemic emphasized that non-frontline workers were more exposed to the risk of vicarious traumatization (12), probably because of inadequately trainined to manage emergencies like epidemics/pandemics. On the contrary, gender was the only demographic variable we found to be significantly associated with all three health outcomes. This is likely due to the fact that the nursing profession is mainly female and 78% of our sample included women. Moreover, gender was highly associated with crying at work due probably to the fact that women usually cry more than men (49).

Lastly, age and caring/non-caring for patients who died of COVID-19 were both significantly associated only with rumination. Regarding age range, our results showed no significant difference in rumination, although it would seem that ruminative thinking increases as age progresses. However, given the contrasting results on the matter presented in literature, it remains unclear how age affects rumination (50). Regarding caring/non-caring for patients who died of COVID-19, the results show that nurses with experience of dead patients had higher levels of rumination. Therefore, nurses may perceive the death of patients whom they cared for during the pandemic as a strong emotional experience that adds excessive pressure, thus leading to rumination.

### Limitation and Future Research

This study presents a few limitations that may be addressed in further research. Firstly, the online system used to collect data may have determined a sampling bias due to the random selection of participants. In this sense, our sample might not be representative of the nursing population and generalizability should be done with caution. A stratified survey would reduce sampling errors and enhance the external validity of studies (51). Secondly, this study lacks longitudinal study design. We carried out a cross-sectional study that does not allow for causal connections between variables (52). While our results are overall consistent with previous studies on epidemics/pandemics, future studies should test long-term effects of the COVID-19 pandemic on nurses' health outcomes. Thirdly, we used a self-administrated questionnaire that has limitations in terms of rating bias. Nevertheless, the health outcomes analyzed in this study (job stress, crying, and rumination) are based on the perception of a discomfort at work during a pandemic. In this sense, self-report questionnaires are adequate instruments to collect perception data. Finally, we chose measure some variables with one item. We are aware that multi-item psychometric scales are more reliable in assuring content validity. Nevertheless, single-item scales can be a good compromise between practical needs and psychometric concerns (53), especially when emergency situations like the pandemics demand to reduce the time needed to complete the survey. Fourthly, we measured some variables by using single items in order to complete the survey in <10 min. This choice was due to the period of high emergency in which the study was conducted (in the middle of the first COVID-19 wave in Italy), such that it was necessary to collect data promptly without cognitively overloading the workers. Although the choice to use single items is questioned as multiple-item scales tend to be more reliable and ensure content validity, it is generally agreed that single-item measures provide an acceptable balance between practical needs and psychometric concerns. They are usually used in occupational health studies and are considered to be reliable (54). Finally, the main measures used in our study were adapted from a previous instrument developed during SARS outbreak. Our study was carried out during the early phase of the pandemic and there was not sufficient time to develop and validate new scales. In this sense, we decided to use a reliable measure from previous studies. However, new scales were developed in the last months and future research could examine the validity of these measures.

### Practical Implications for Nurses' Health

Despite the limitations, this study can have important implications for nurses. Nurses' work-related and non-work-related worries about the pandemic could affect their overall effectiveness at work. Therefore, these concerns should be addressed by devising effective preventive strategies to avoid prolonged consequences in terms of mental health. Among the interventions to reduce nurses' worries, providing a place where they can temporarily isolate themselves from their family (55) may be an effective strategy. In addition, as stress theory revealed (56, 57), workload represents a crucial stressor for professionals. It should thus be reduced by increasing human resources and providing organizational support to limit the negative impact in terms of stress and rumination. Moreover, as crying at work is associated with both higher levels of job stress and rumination, due probably to excessive demands and emotional pressures during the pandemic, health organizations should implement actions to reduce stress and foster psychological support especially for nurses with inadequate training in emotion regulation labor. Finally, as rumination is associated with a number of stress-related disorders, it would be important to reduce ruminative thinking about the pandemic through coping strategies which helpnurses to recover during leisure time and reduce job stress (58).

## Conclusions

The psychological impact that the COVID-19 pandemic may cause in the medium/long term could be greater than the economic one. This is the main challenge that health organizations will have to face in the future: in fact, we are currently experiencing the third wave of this outbreak. In this phase, it is crucial that decision-makers develop awareness of the impact of this pandemic on nurses' mental health and promptly implement regional and national interventions to lessen the risk of developing depressive symptoms and post-traumatic stress disorders.

## Data Availability Statement

The raw data supporting the conclusions of this article will be made available by the authors upon reasonable request.

## Ethics Statement

The study complies with the Declaration of Helsinki and with the General Data Protection Regulation (EU) 2016/679 (GDPR). Participation was voluntary and anonymous, according to Italian Data protection law (e.g., Decree n. 196/2003). Participants could interrupt their participation in the survey at any time without any adverse consequence. We consulted the Institutional Review Boards of the University of Cagliari, which informally said that Ethical review and approval was not required for the study on human participants, in accordance with the local legislation and institutional requirements.

## Author Contributions

MG, IPi, and IPo conceived and designed the study, collected, analyzed and interpreted the data, and drafted the manuscript. FM analyzed the data, edited, and revised the manuscript. GF, ED'A, PC, and MC edited and revised the manuscript critically. All authors approved the final version of the manuscript.

## Conflict of Interest

The authors declare that the research was conducted in the absence of any commercial or financial relationships that could be construed as a potential conflict of interest.
